# Pulvinar signal abnormalities on MRI are associated with atraumatic hip pain

**DOI:** 10.1007/s00256-026-05169-4

**Published:** 2026-02-20

**Authors:** Adrian A. Marth, Brian Tangsombatvisit, Zehra Akkaya, Gabby B. Joseph, Alan L. Zhang, Richard B. Souza, Thomas M. Link

**Affiliations:** 1 Department of Radiology and Biomedical Imaging, University of California, San Francisco, 185 Berry Street, San Francisco, CA 94107 USA; 2 Department of Radiology, Ankara University, Ankara, Turkey; 3 Department of Orthopaedic Surgery, University of California, San Francisco, San Francisco, USA; 4 Department of Physical Therapy and Rehabilitation Science, University of California, San Francisco, San Francisco, CA USA

**Keywords:** MRI, Hip, Pulvinar, Pain, Grading system

## Abstract

**Objective:**

To investigate MRI abnormalities of the pulvinar and ligamentum teres (LT) in patients with atraumatic hip pain, as well as to describe a grading system for pulvinar signal abnormalities and test its reproducibility.

**Materials and methods:**

This retrospective study included 128 patients with atraumatic hip pain and no reported structural abnormalities other than those of the pulvinar and LT on hip MRI, along with 64 asymptomatic controls. MR images were evaluated by two readers and included grading of pulvinar signal abnormalities (normal, grade 1: < 50% fat replacement, grade 2: > 50% fat replacement), LT signal abnormality, LT thickening, and LT tear. Ordinal variables were dichotomized for further analysis. Group differences were analyzed using logistic regression models. Inter-reader agreement was assessed using kappa statistics.

**Results:**

Pulvinar signal abnormalities were significantly associated with a higher odds ratio (OR) of hip pain (OR, 4.28; 95% CI, 1.81–11.49; *p* = 0.002). No significant group differences were found for LT signal increase, LT thickening, or LT tear (*p*-value range = 0.43–0.71). Inter-reader and intra-reader agreement for pulvinar signal abnormalities grading were almost perfect (*κ* = 0.85 [95% CI, 0.76–0.93] and *κ* = 0.92 [95% CI, 0.64–1.00]).

**Conclusion:**

Patients with atraumatic hip pain undergoing MRI demonstrated a significantly higher prevalence of pulvinar signal abnormalities in the absence of other relevant structural pathology. These results underscore the importance of systematically evaluating the pulvinar during hip MRI interpretation. Furthermore, a grading system for pulvinar signal abnormalities was introduced.

## Introduction

In patients with hip pain with inconclusive findings from physical examination and negative radiographs, MRI with or without intra-articular contrast may be used to assess osseous and soft-tissue pathologies in or around the joint [1]. However, a subset of patients with atraumatic hip pain demonstrates no structural imaging abnormalities; conversely, the significance of some imaging abnormalities, including isolated acetabular labral tears and gluteal tendinopathy, is unclear, as these findings are frequently encountered in asymptomatic individuals [2–5].

The acetabular fossa is a region of the hip joint that has received limited attention in the literature, particularly regarding its potential relation to hip pain [6]. It contains two primary structures: the first is the pulvinar, [6, 7] , an intra-articular fat pad covered by synovium which contains intra-articular adipose tissue. Intra-articular adipose tissue is anatomically and molecularly distinct from subcutaneous and visceral fat, with unique cellular composition, histologic architecture, and gene-expression profiles [6]. Functionally, it contributes to joint homeostasis through adipokine secretion and serves as a reservoir of mesenchymal stem cells that may participate in tissue repair [8]. However, its role in relation to hip pain is currently not well understood. The second structure in the acetabular fossa is the ligamentum teres, which merges with the periosteum of the fovea capitis, the transverse acetabular ligament, and the bony margins of the acetabular notch. Once considered a vestigial structure, interest in the ligamentum teres has increased with the advent of hip arthroscopy [9]. Tears of the ligamentum teres have been associated with hip pain, particularly in the presence of synovitis, or in athletes with a history of trauma [10, 11]. Ligamentum teres tears can be graded using the Gray and Villar classification system [12], whereas for pulvinar abnormalities, no classification system currently exists.

Given the lack of knowledge concerning the clinical significance of imaging abnormalities of the acetabular fossa, the primary aim of this study was to evaluate MRI findings of the pulvinar and the ligamentum teres in patients with atraumatic hip pain but without reports of other relevant intra- or extra-articular structural abnormalities, and to compare these findings with a group of asymptomatic controls. The secondary aim was to develop an MRI-based grading system for pulvinar signal abnormalities and to evaluate its reproducibility.

## Materials and methods

### Study design and cohort selection

**Fig. 1:**
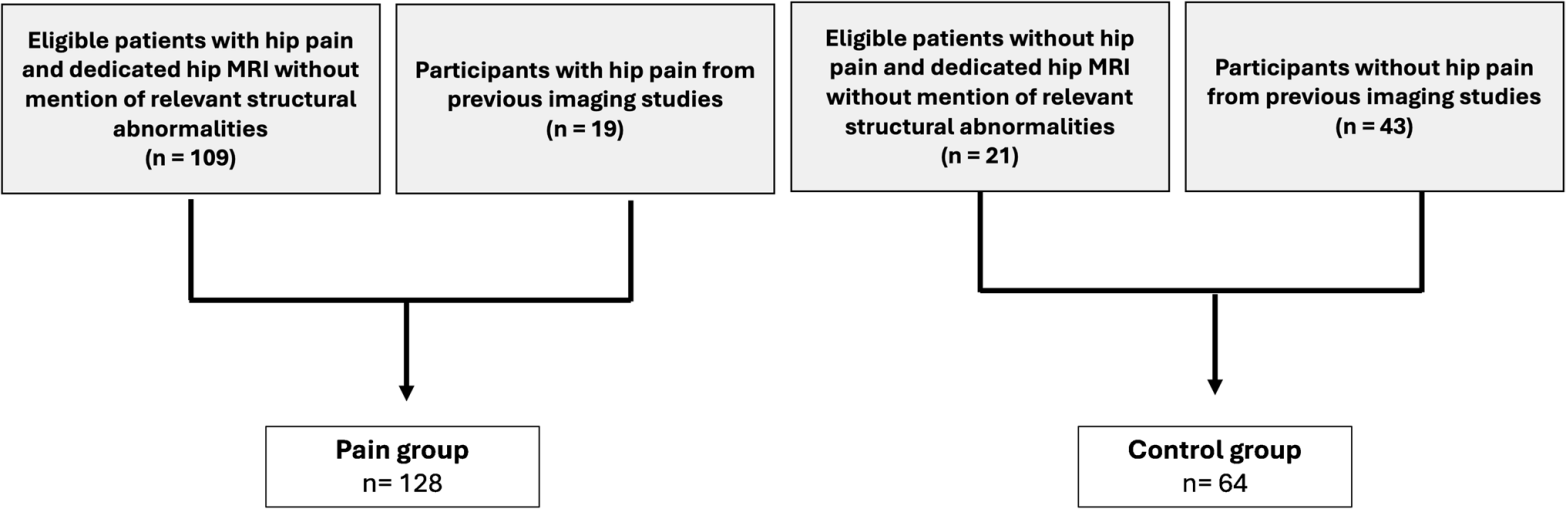
Subject selection from a natural language processing–based institutional MRI database search and previously conducted studies

This retrospective study was approved by the institutional review board, which waived the requirement for written informed consent. MRI reports from the institutional database were retrieved using a natural language processing–based report search tool (mPower, Nuance Communications Inc). Corresponding clinical information was drawn from the electronic health record system (Epic Systems Corporation). The search criteria included patients who underwent dedicated hip MRI without intra-articular contrast for evaluation of unilateral atraumatic hip pain between 2012 and 2025 without mention of structural abnormalities in the MRI reports. Medical records of the orthopedic department were screened to confirm that the clinical exam suggested that pain was originating from the hip joint. To increase the sample size and avoid confounding related to differences in imaging protocols, two additional groups were included for the present study. First, individuals from previous imaging studies investigating quantitative imaging techniques to detect progression of early hip osteoarthritis, the relationship between ligamentum teres lesions and hip osteoarthritis, and hip capsule morphology in patients with hip pain [13–15] were included. Participants from these studies with hip pain (Hip disability and Osteoarthritis Outcome Scores [Pain Subscale] of ≤ 70 [16, 17] or hip pain in the clinical records) were included in the pain group, whereas those without hip pain (Hip disability and Osteoarthritis Outcome Scores [Pain Subscale] = 100 or no hip pain in the clinical records) were included in the control group. Second, patients without hip pain who underwent unilateral clinical hip MRI between 2023 and 2025, e.g., for incidental bone lesions on radiographs, were searched from the institutional database as described above. The subject selection process is illustrated in Fig. 1.

Eligibility criteria for the pain and the control group included the following: (i) age ≥ 18 years, (ii) no other potential causes for hip pain (e.g., low back pain, inguinal hernia), (iii) no relevant intra-articular structural abnormalities on hip MRI (defined as Scoring Hip Osteoarthritis with MRI (SHOMRI [18]) scores < 1 for articular cartilage, subchondral cysts, joint effusion, labral tears, bone marrow lesions), (iv) no relevant extra-articular structural abnormalities on hip MRI (e.g., tear of the gluteus medius/minimus tendon, signs of bursitis, iliopsoas tendon pathology, ischiofemoral impingement), (v) no relevant osseous abnormalities on plain radiographs or MRI (acetabular retroversion, coxa profunda, acetabular protrusion, femoroacetabular impingement morphology, or signs of developmental dysplasia of the hip), (vi) no history of inflammatory arthropathy, (vii) no previous history of hip surgery, (viii) no hip trauma in the last six months, (ix) no history of professional athletic activity.

### Image acquisition parameters

Patients from the pain and the control group underwent unilateral hip MRI using a 3 Tesla scanner (GE Medical Systems Discovery MR750, GE Healthcare) equipped with an 8-channel flexible coil (GE Healthcare) positioned over the hip. The protocol comprised tri-planar 2D fat-suppressed, fluid-sensitive fast spin-echo sequences in coronal, sagittal, and oblique axial orientations (field of view [FOV] = 18.0 × 18.0 cm; repetition time [TR] = 2400–3700 ms; echo time [TE] = 60 ms; slice thickness = 4.0 mm). In addition, a coronal T1-weighted fast spin-echo sequence (FOV = 18.0 × 18.0 cm; TR = 516–625 ms; TE = 8 ms; slice thickness = 4.0 mm) was obtained. The participants from the previous studies [13, 14] underwent unilateral hip MRI on a 3 Tesla scanner (GE Healthcare) using an 8-channel receive-only cardiac coil (GE Healthcare). The protocol included intermediate-weighted, fat-suppressed fast spin-echo sequences in three planes: (1) sagittal (TR 3678 ms; TE 60 ms; slice thickness 4 mm; FOV 14 cm), (2) oblique coronal (TR 2496 ms; TE 60 ms; slice thickness 4 mm; FOV 20 cm), and (3) oblique axial (TR 2800 ms; TE 60 ms; slice thickness 3 mm; FOV 18 cm).

### Image analysis and pulvinar grading system

Image readings were performed independently by two board-certified fellowship-trained musculoskeletal radiologists (TML) with five and more than 20 years of experience with musculoskeletal MRI. The primary outcomes of interest included pathological changes of the pulvinar and the ligamentum teres. For the pulvinar, a grading system was developed to assess signal abnormalities on coronal T1-weighted images and/or coronal intermediate-weighted fat-suppressed images. Grade 0 was defined as normal pulvinar signal, characterized by homogenous hyperintense fat signal on T1-weighted images and the absence of diffuse hyperintense signal on fat-suppressed fluid-sensitive images. Grade 1 was defined as T1 dark/T2 bright signal abnormalities with greater 50% fat replacement and Grade 2 as signal abnormalities with 50% and greater fat replacement. The grading system was intentionally designed to be simple, reproducible, and applicable to routine clinical MRI. For ligamentum teres tears, the Gray and Villar classification [12] was used (intact, partial tear, complete tear). Furthermore, the presence of signal increase and/or thickening of the ligament teres was assessed. Readers were blinded to demographic and clinical data and analyzed the images in random order on the local PACS viewer (Visage v7, Visage Imaging Inc.). Two initial consensus training sessions were performed before image readings were started. In cases of discrepant assessments for the primary outcomes, consensus readings were performed. Intra-reader reliability was performed four weeks after initial imaging by one reader (AAM) for a subset of 20 random subjects.

### Statistical analysis

Statistical analyses were performed using R version 4.5.0 (R Foundation for Statistical Computing). Group differences in demographic variables were assessed using the Wilcoxon rank-sum test for continuous variables and the chi-square test for categorical variables. Logistic regression models were used to evaluate the associations between MRI-based predictors and the presence of pain (pain group vs. control group), which were adjusted for age, sex, and BMI. The predictors were defined as follows: for the pulvinar, the presence or absence of signal abnormalities and for the ligamentum teres, presence or absence of signal increase, thickening, or tear (partial or complete) was evaluated. The rationale for dichotomization of the exposures was that diagnostic accuracy of grading pulvinar signal abnormalities has not yet been described, and only limited data on the diagnostic accuracy of unenhanced MRI for grading ligamentum teres tears exists [19]. The associations of the two grades of pulvinar signal abnormalities with atraumatic hip pain were evaluated separately as a sensitivity analysis. Inter- and intra-reader reliability for all outcomes was assessed using squared weighted Cohen’s kappa statistics. Level of agreement was reported according to Landis et al. [20].

## Results

### Patient demographics and pain features

A total of 128 patients with atraumatic hip pain and 64 controls were included in the analysis. Group characteristics are summarized in Table 1. There were no significant differences between the two groups for age (*p* = 0.07), sex (*p* = 0.25), or BMI (*p* = 0.27). Pain duration data were available for 100 patients, with a mean duration of 29.7 ± 51.9 months. With respect to pain location, the most frequently reported sites were the lateral hip (*n* = 26, 30.2%), anterior hip (*n* = 19, 22.1%), groin (*n* = 16, 18.6%), and posterior hip (*n* = 14, 16.3%). Diffuse pain involving more than one site was reported by 11 patients (12.8%). In 42 patients, hip pain without specific pain location was reported.

**Table 1:** Demographic data of the study subjects by group. Data are given as mean ± standard deviation for continuous data and as frequency with percentage in parentheses for categorical data

	**Pain group** **(*n* = 128)**	**Control group** **(*n* = 64)**	**Overall** **(*n* = 192)**	***p*-value**
**Age**	39.5 ± 11.7	43.4 ± 14.5	40.8 ± 12.4	0.07
**Sex**				0.25
Female	92 (71.9)	40 (62.5)	132 (68.8)	
Male	36 (28.1)	24 (37.5)	60 (31.2)	
**BMI**	25.2 ± 5.4	25.6 ± 4.2	25.3 ± 5.0	0.27

### Imaging findings

**Fig. 2:**
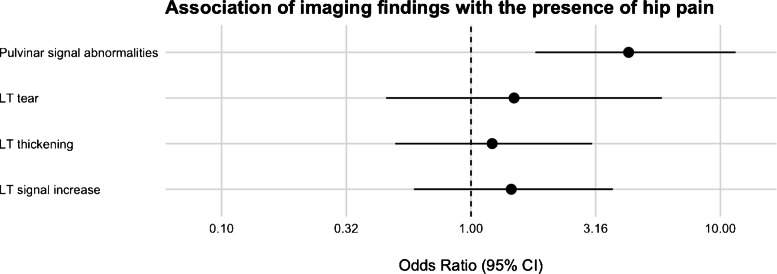
Associations of imaging findings with the presence of hip pain. A significant association was found for pulvinar signal abnormalities (OR, 4.28, 95% CI, 1.81–11.49; *p* = 0.002), whereas no significant differences between the groups were found for ligamentum teres signal increase (OR, 1.44; 95% CI, 0.59–3.68; *p* = 0.43), ligamentum teres tear (OR, 1.42; 95% CI, 0.43–5.60; *p* = 0.59), or for ligamentum teres thickening (OR, 1.19; 95% CI, 0.48–3.00; *p* = 0.71). The ORs for grade 2 abnormalities showed wide confidence intervals, reflecting the limited number of cases in the control group (*LT*, Ligamentum teres)

**Fig. 3:**
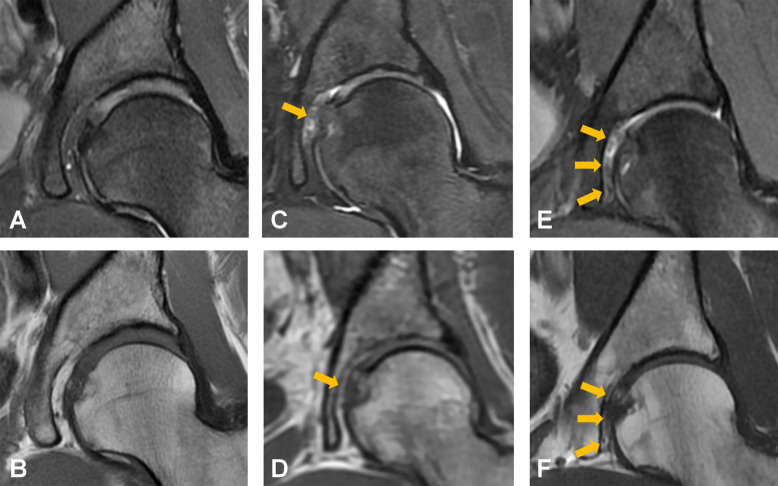
Grading of pulvinar signal abnormalities on coronal T1-weighted and intermediate-weighted fat-suppressed images. Grade 0 (**A**, **B**): normal appearance of the pulvinar. Grade 1 (**C**, **D**): centrally located pulvinar signal abnormalities with < 50% fat replacement (arrows). Grade 2 (**E**, **F**): extensive pulvinar signal abnormalities with > 50% fat replacement (arrows)

**Fig. 4:**
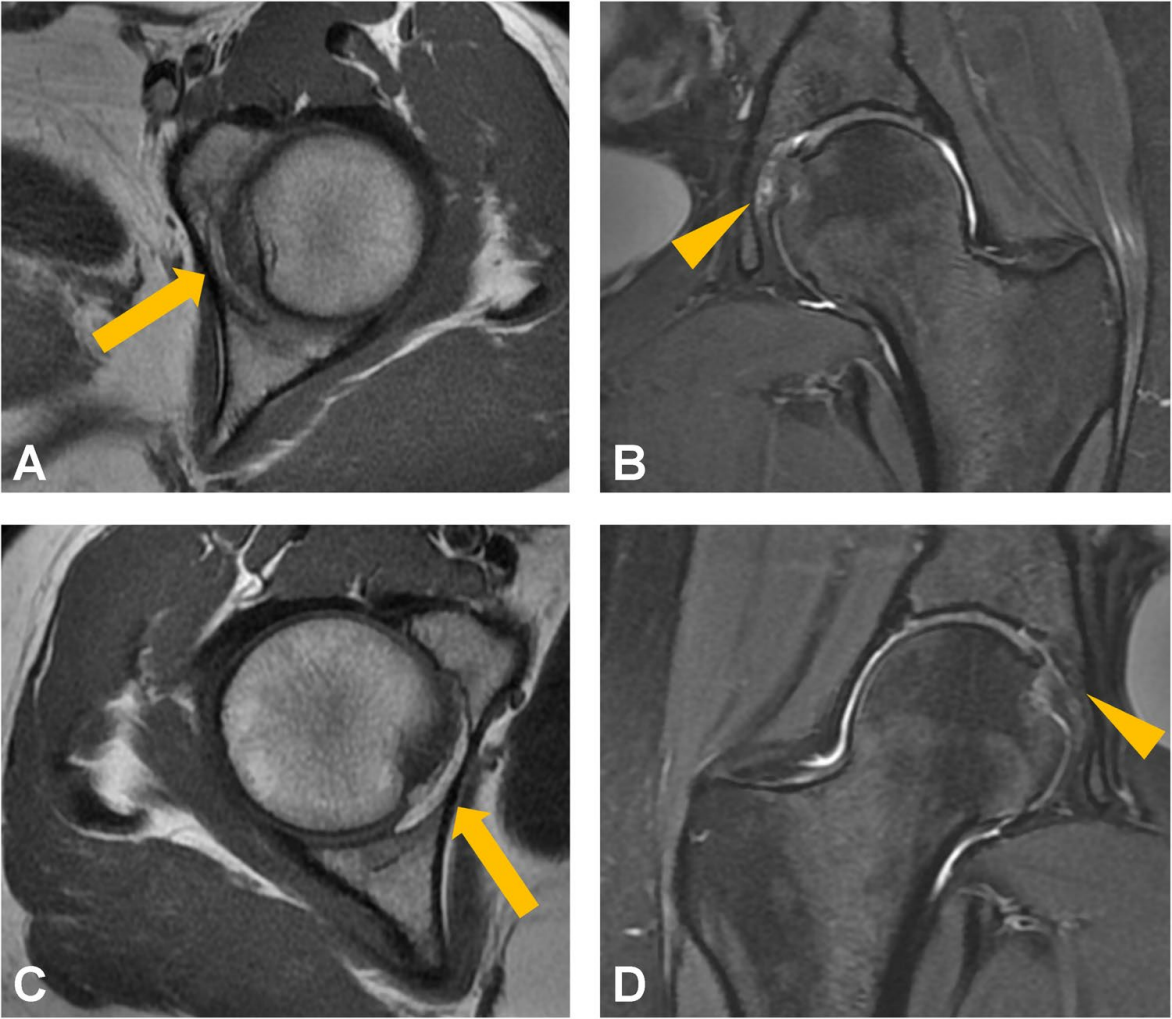
Representative transverse T1-weighted and coronal fat-suppressed T2-weighted images of a 34-year-old woman presenting with atraumatic left-sided hip pain for 24 months. Pulvinar signal abnormalities with central replacement of fat signal (total < 50%, grade 1) is marked by the arrow in (**A**). Centrally located focal signal increase is highlighted by the arrowhead in (**B**). Note the unremarkable pulvinar of the right side in the same patient (arrow, **C**; arrowhead, **D**)

**Fig. 5:**
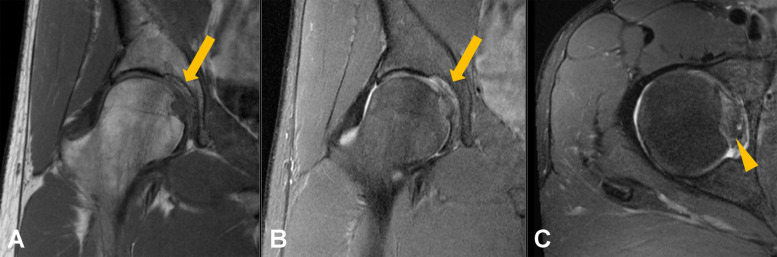
Representative coronal T1-weighted images (**A**), coronal fat-suppressed T2-weighted images (**B**), and transverse fat-suppressed T2-weighted images (**C**) of a 42-year-old man presenting with atraumatic right-sided hip pain for 8 months. Signal abnormalities of the pulvinar replacing more than 50% of the fat pad (grade 2) are highlighted by arrows in **A** and **B**. Note the concomitant signal abnormalities and thickening of the ligamentum teres (arrowhead, **C**)

**Fig. 6:**
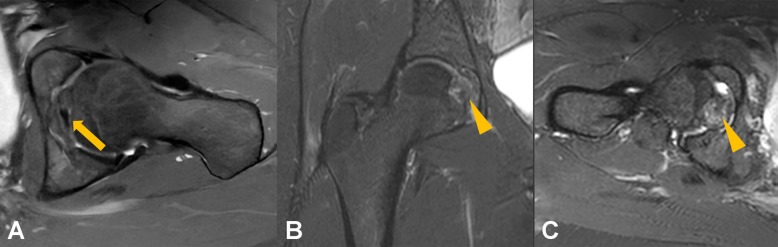
Representative coronal and transverse fat-suppressed T2-weighted images (**A**, **B**) of a 31-year-old woman without hip pain (**A**) and a 33-year-old woman presenting with atraumatic hip pain (**B**, **C**). Thickening of the ligamentum teres was observed in the asymptomatic control (arrow, **A**). The notable signal increase and thickening of the ligamentum teres in the patient with hip pain are marked by the arrowheads in (**B**, **C**)

A descriptive summary of imaging findings is presented in Table 2. After MRI review, congenital absence of the ligamentum teres was identified in four patients in the pain group and two subjects in the control group; these individuals were excluded from the final analysis. The presence of pulvinar signal abnormalities was associated with a significantly higher odds of hip pain (OR, 4.28, 95% CI, 1.81–11.49; *p* = 0.002). In a sensitivity analysis, the association of both grades of pulvinar signal abnormalities remained significant when evaluated separately (grade 1: OR, 3.38; 95% CI, 1.33–9.93; *p* = 0.0016; grade 2: OR, 8.42; 95% CI, 1.52–158.22; *p* = 0.0047). On the other hand, no significant differences between the groups were found for ligamentum teres signal increase (OR, 1.44; 95% CI, 0.59–3.68; *p* = 0.43), ligamentum teres tear (OR, 1.42; 95% CI, 0.43–5.60; *p* = 0.59), or for ligamentum teres thickening (OR, 1.19; 95% CI, 0.48–3.00; *p* = 0.71). A forest plot of ORs is displayed in Fig. 2. The proposed grading system for pulvinar signal abnormalities is illustrated in Fig. 3. Representative images of pulvinar and ligamentum teres abnormalities are presented in Figs. 4, 5, and 6.

**Table 2:** MRI findings by groups. Data are given as frequency with percentage in parentheses

	**Pain group** **(*n* = 128)**	**Control group** **(*n* = 64)**	**Overall** **(*n* = 192)**
**Ligamentum teres**			
Intact	110 (85.9)	58 (90.6)	168 (87.5)
Partial tear	13 (10.2)	4 (6.2)	17 (8.9)
Complete tear	1 (0.8)	–	1 (0.5)
Congenital absence	4 (3.1)	2 (3.1)	6 (3.1)
Thickening	32 (25.0)	13 (20.3)	45 (23.4)
Signal increase	30 (23.4)	12 (18.8)	42 (21.9)
**Pulvinar**			
Normal pulvinar signal	84 (65.6)	55 (85.9)	139 (72.4)
Signal abnormalities with < 50% fat replacement	30 (23.4)	7 (10.9)	37 (19.3)
Signal abnormalities with ≥ 50% fat replacement	14 (10.9)	2 (3.1)	16 (8.3)

### Reproducibility of the pulvinar grading system and other outcomes

The pulvinar grading system demonstrated almost perfect inter-reader agreement of *κ* = 0.85 (95% CI, 0.76–0.93) and almost perfect intra-reader agreement of *κ* = 0.92 (95% CI, 0.64–1.00). Inter-reader agreement for the other primary outcomes was substantial to almost perfect: it was *κ* = 0.78 (95% CI, 0.68–0.87) for ligamentum teres signal increase, *κ* = 0.92 (95% CI, 0.85–0.97) for ligamentum teres thickening, and *κ* = 0.68 (95% CI, 0.49–0.85) for ligamentum teres tear. Intra-reader agreement for the other primary outcomes, assessed in a subset of 20 subjects, ranged from perfect agreement for ligamentum teres signal increase (*κ* = 1.00) to almost perfect for ligamentum teres thickening (*κ* = 0.86 [95% CI, 0.68–1.00]) and ligamentum teres tear (*κ* = 0.89 [95% CI, 0.61–1.00]).

## Discussion

This study evaluated pathological changes of the pulvinar and ligamentum teres in patients with atraumatic hip pain and no soft-tissue or osseous abnormalities on MR images. Pulvinar signal abnormalities were associated with hip pain, while no differences between the two groups were found for signal increase, thickening, or tear of the ligamentum teres. The grading system of pulvinar signal abnormalities demonstrated high inter-and intra-reader agreement.

The association of pulvinar signal abnormalities with hip pain is a particularly interesting finding, as the relationship between these pulvinar findings and pain has not been previously explored. In the current literature, the pulvinar has been characterized as a “canary in a coal mine” [21], advocating that early cartilage and labral injury in the setting of osteoarthritis may initially manifest within the acetabular fossa. This concept is supported by the observation that osteophyte formation originates centrally within the acetabular fossa and is associated with loss of pulvinar tissue [22].

Although causality between pulvinar signal abnormalities and hip pain cannot be inferred from this study, our results support a model in which the pulvinar acts as an important indicator of hip pathology and pain: it contributes to joint homeostasis under physiologic conditions, but shifts toward an inflammatory, pain-associated state early in degeneration. The hypothesis that these signal abnormalities represent synovitis would align with Byrd’s proposal that synovial metaplasia of the pulvinar can serve as a primary pain generator [23], given the observation that intra-articular adipose tissue also exhibits nociceptive innervation [24]. Furthermore, our findings would be supported by recent studies investigating the infrapatellar fat pad in the knee, which found that intra-articular adipose tissue can adopt a pro-inflammatory profile, with increased production of catabolic proteins into the synovial fluid that may accelerate disease progression in osteoarthritis [25–27].

On the other hand, these signal abnormalities may also be a secondary finding related to hip microinstability, given the concomitant high prevalence of ligamentum teres abnormalities (up to 25%) compared to prior arthroscopic reports (4–15% [28, 29]). The ligamentum teres serves as a secondary stabilizer of the capsular ligaments, resisting subluxation of the femoral head through a sling-like mechanism, resembling the functional properties of the anterior cruciate ligament in the knee [10, 30]. Consequently, injury of the ligament results in altered joint biomechanics, which is associated with damage to the labrum and articular cartilage [14, 31, 32]. Future studies are warranted to investigate the longitudinal relationship between pulvinar signal abnormalities and degenerative changes of the hip. Such an association appears biologically plausible, as similar pathophysiologic patterns have been observed for the infrapatellar fat pad in the knee [33, 34].

On the other hand, no significant association between ligamentum teres abnormalities and hip pain was found in this cohort. However, associations of ligamentum teres tears with pain are predominantly reported for complete tears or avulsion injuries in athletes and/or after trauma [11, 28, 31], which would be consistent with our findings, as only patients without a history of trauma or professional athletic activity were included. As one of the first studies to systematically characterize pulvinar signal abnormalities on hip MRI, our findings have led to a more deliberate evaluation of the acetabular fossa and increased recognition of this imaging finding in clinical practice at our institution.

The proposed grading system of pulvinar signal abnormalities yielded almost perfect inter- and intra-reader reliability. We developed this system as it is easy to implement and comprised of only three grades (normal signal, grade 1 = less than 50% fat replacement, grade 2 = more than 50% fat replacement). Grading can be performed on standard coronal and transversal T1-weighted and fat-suppressed, intermediate-weighted fluid-sensitive images. Both grades of pulvinar signal abnormalities were significantly associated with hip pain. However, the lack of grade 2 abnormalities in the control group resulted in wide confidence intervals, which require these findings to be interpreted with caution.

The following limitations of this study have to be acknowledged: First, its retrospective design precluded the use of histopathology as a reference standard to verify imaging findings. Second, although we observed significant associations between morphological features and symptoms, our findings do not allow us to extrapolate biomechanical causality, which could be investigated in a future prospective study. Third, imaging relied exclusively on non-contrast MRI, which may constrain reliability and reproducibility, particularly for evaluation of the ligamentum teres. Fourth, the sequences of the MRI protocols had a slice thickness of up to 4 mm, which may limit sensitivity for detecting pathologies of intra-articular structures. Fifth, surgical follow-up data were not available for the vast majority of patients in this retrospective cohort, which precluded meaningful surgical correlation of pulvinar MRI findings. Future prospective studies with dedicated surgical documentation will be necessary to address this limitation; however, MRI findings may still be non-specific despite surgical correlation. The association of pain location with pulvinar signal abnormalities was not assessed, as pain was heterogeneous, frequently non-specific, and incompletely documented in a substantial proportion of patients. Finally, because the cohort excluded subjects with trauma and/or professional athletes and was derived from a single institution, the generalizability of our findings remains to be confirmed. Future studies including patients with concomitant intra-articular abnormalities may help elucidate potential interactions between pulvinar signal abnormalities and other sources of hip pathology and further clarify their contribution to hip pain.

In conclusion, this study found an association between pulvinar signal abnormalities and atraumatic hip pain in a cohort of patients without other structural abnormalities on MRI, while no such association was found for ligamentum teres pathologies. These results highlight the pulvinar's importance in patients with atraumatic hip pain and emphasize the need for rigorous MRI assessment, particularly when no other hip pathology is visible.

## Data Availability

Data are available from the corresponding author upon reasonable request.

## References

[1] Reiman MP, Agricola R, Kemp JL, Heerey JJ, Weir A, Klij P, et al. Consensus recommendations on the classification, definition and diagnostic criteria of hip-related pain in young and middle-aged active adults from the International Hip-related Pain Research Network, Zurich 2018. Br J Sports Med. 2020;54:631–41. 10.1136/bjsports-2019-101453. 10.1136/bjsports-2019-101453 31959678

[2] Frank JM, Harris JD, Erickson BJ, Slikker W, Bush-Joseph CA, Salata MJ, et al. Prevalence of femoroacetabular impingement imaging findings in asymptomatic volunteers: a systematic review. Arthroscopy: The Journal of Arthroscopic & Related Surgery. 2015;31:1199–204. 10.1016/j.arthro.2014.11.042. 25636988 10.1016/j.arthro.2014.11.042

[3] Kumar D, Wyatt C, Chiba K, lee S, Nardo L, Link TM, et al. Anatomic correlates of reduced hip extension during walking in individuals with mild-moderate radiographic hip osteoarthritis. J Orthop Res. 2015;33:527–34. 10.1002/jor.22781. 25678302 10.1002/jor.22781 PMC4376613

[4] Meghpara MB, Bheem R, Shah S, Shapira J, Maldonado DR, Rosinsky PJ, et al. Prevalence of gluteus medius pathology on magnetic resonance imaging in patients undergoing hip arthroscopy for femoroacetabular impingement: asymptomatic tears are rare, whereas tendinosis is common. Am J Sports Med. 2020;48:2933–8. 10.1177/0363546520952766. 32881581 10.1177/0363546520952766

[5] Heerey JJ, Kemp JL, Mosler AB, Jones DM, Pizzari T, Souza RB, et al. What is the prevalence of imaging-defined intra-articular hip pathologies in people with and without pain? A systematic review and meta-analysis. Br J Sports Med. 2018;52:581–93. 10.1136/bjsports-2017-098264. 10.1136/bjsports-2017-098264 29540366

[6] Slullitel PA, Coutu D, Buttaro MA, Beaule PE, Grammatopoulos G. Hip preservation surgery and the acetabular fossa: a canary in a coal mine? Bone Jt Res Bone & Joint. 2020;9:857–69. 10.1302/2046-3758.912.BJR-2020-0254.R1. 10.1302/2046-3758.912.BJR-2020-0254.R1 PMC9021901 33275027

[7] Cerezal L, Kassarjian A, Canga A, Dobado MC, Montero JA, Llopis E, et al. Anatomy, biomechanics, imaging, and management of ligamentum teres injuries. Radiographics. 2010;30:1637–51. 10.1148/rg.306105516. 10.1148/rg.306105516 21071380

[8] Labusca L, Zugun-Eloae F, Frontiers. The unexplored role of intra-articular adipose tissue in the homeostasis and pathology of articular joints. Front Vet Sci. 2018. 10.3389/fvets.2018.00035. 29556503 10.3389/fvets.2018.00035 PMC5845097

[9] Botser IB, Martin DE, Stout CE, Domb BG. Tears of the ligamentum teres: prevalence in hip arthroscopy using 2 classification systems. Am J Sports Med. 2011;39:117S-S125. 10.1177/0363546511413865. 21709041 10.1177/0363546511413865

[10] O’Donnell JM, Arora M. A novel and simple classification for ligamentum teres pathology based on joint hypermobility. J Hip Preserv Surg. 2018;5:113–8. 10.1093/jhps/hnx039. 29876126 10.1093/jhps/hnx039 PMC5961003

[11] Abel F, Schmaranzer F, Sutter R. Sports-related Hip Injuries. Semin Musculoskelet Radiol. 2025;29:442–56. 10.1055/s-0045-1805079. 40393502 10.1055/s-0045-1805079

[12] Gray AJR, Villar RN. The ligamentum teres of the hip: an arthroscopic classification of its pathology. Arthroscopy: The Journal of Arthroscopic & Related Surgery. 1997;13:575–8. 10.1016/S0749-8063(97)90182-1. 9343644 10.1016/s0749-8063(97)90182-1

[13] Gallo MC, Wyatt C, Pedoia V, Kumar D, Lee S, Nardo L, et al. T1ρ and T2 relaxation times are associated with progression of hip osteoarthritis. Osteoarthritis Cartilage. 2016;24:1399–407. 10.1016/j.joca.2016.03.005. 26973330 10.1016/j.joca.2016.03.005 PMC4955678

[14] Akkaya Z, Giesler PJ, Roach KE, Joseph GB, McCulloch CE, Bharadwaj UU, et al. Ligamentum teres lesions are associated with compositional and structural hip cartilage degenerative change: region-specific cartilage degeneration. Eur Radiol. 2025;35:2275–86. 10.1007/s00330-024-11030-w. 39177856 10.1007/s00330-024-11030-w

[15] Luitjens J, Gassert FG, Patwardhan V, Bhattacharjee R, Joseph GB, Zhang AL, et al. Is hip capsule morphology associated with hip pain in patients without another structural correlate? Eur Radiol. 2024;34:4321–30. 10.1007/s00330-023-10307-w. 38170264 10.1007/s00330-023-10307-w PMC11213662

[16] Larsen P, Rathleff MS, Roos EM, Elsoe R. National population-based reference data for the Hip Disability and Osteoarthritis Outcome Score (HOOS). Arch Orthop Trauma Surg. 2023;143:6865–74. 10.1007/s00402-023-04915-w. 37277643 10.1007/s00402-023-04915-w PMC10542294

[17] Emara AK, Pasqualini I, Jin Y, Klika AK, Orr MN, Rullán PJ, et al. What are the diagnosis-specific thresholds of minimal clinically important difference and patient acceptable symptom state in hip disability and osteoarthritis outcome score after primary total hip arthroplasty? J Arthroplasty. 2024;39:1783-1788.e2. 10.1016/j.arth.2024.01.051. 38331359 10.1016/j.arth.2024.01.051

[18] Lee S, Nardo L, Kumar D, Wyatt CR, Souza RB, Lynch J, et al. Scoring hip osteoarthritis with MRI (SHOMRI): a whole joint osteoarthritis evaluation system. J Magn Reson Imaging. 2015;41:1549–57. 10.1002/jmri.24722. 25139720 10.1002/jmri.24722 PMC4336224

[19] Shakoor D, Farahani SJ, Hafezi-Nejad N, Johnson A, Vaidya D, Khanuja HS, et al. Lesions of ligamentum teres: diagnostic performance of MRI and MR arthrography-a systematic review and meta-analysis. AJR Am J Roentgenol. 2018;211:W52-63. 10.2214/AJR.17.19198. 29792743 10.2214/AJR.17.19198

[20] Landis JR, Koch GG. The measurement of observer agreement for categorical data. Biometrics. 1977;33:159–74. 843571

[21] Byrd JWT. Synovial disease and sepsis. In: Byrd JWT, editor. Oper Hip Arthrosc [Internet]. New York, NY: Springer; 2013 [cited 2025 Aug 18]. p. 203–14. 10.1007/978-1-4419-7925-4_16

[22] Noguchi Y, Miura H, Takasugi S, Iwamoto Y. Cartilage and labrum degeneration in the dysplastic hip generally originates in the anterosuperior weight-bearing area: an arthroscopic observation. Arthroscopy: The Journal of Arthroscopic & Related Surgery. 1999;15:496–506. 10.1053/ar.1999.v15.015049. 10424553 10.1053/ar.1999.v15.015049

[23] Thomas Byrd JW. Indications and contraindications. In: Oper Hip Arthrosc. Springer; 2005. p. 6–35.

[24] Saxler G, Löer F, Skumavc M, Pförtner J, Hanesch U. Localization of SP- and CGRP-immunopositive nerve fibers in the hip joint of patients with painful osteoarthritis and of patients with painless failed total hip arthroplasties. Eur J Pain. 2007;11:67–74. 10.1016/j.ejpain.2005.12.011. 16460974 10.1016/j.ejpain.2005.12.011

[25] Favero M, El-Hadi H, Belluzzi E, Granzotto M, Porzionato A, Sarasin G, et al. Infrapatellar fat pad features in osteoarthritis: a histopathological and molecular study. Rheumatology. 2017;56:1784–93. 10.1093/rheumatology/kex287. 28957567 10.1093/rheumatology/kex287

[26] Felson DT, Niu J, Neogi T, Goggins J, Nevitt MC, Roemer F, et al. Synovitis and the risk of knee osteoarthritis: the MOST study. Osteoarthritis Cartilage. 2016;24:458–64. 10.1016/j.joca.2015.09.013. 26432512 10.1016/j.joca.2015.09.013 PMC4761323

[27] Wagner JG, Chen L, Jiang F, Nedley E, Akkaya Z, Ngarmsrikan C, et al. Relationships between the infrapatellar fat pad and patellofemoral joint osteoarthritis differ with body mass index and sex. J Orthop Res. 2025;43:770–9. 10.1002/jor.26048. 39833110 10.1002/jor.26048 PMC13007260

[28] Byrd JWT, Jones KS. Traumatic rupture of the ligamentum teres as a source of hip pain. Arthroscopy: The Journal of Arthroscopic & Related Surgery. 2004;20:385–91. 10.1016/j.arthro.2004.01.025. 15067278 10.1016/j.arthro.2004.01.025

[29] Bardakos NV, Villar RN. The ligamentum teres of the adult hip. J Bone Joint Surg Br. 2009;91:8–15. 10.1302/0301-620X.91B1.21421. 19091998 10.1302/0301-620X.91B1.21421

[30] Guimarães JB, Arruda PHC, Cerezal L, Ratti MAS, Cruz IAN, Morimoto LR, et al. Hip microinstability: new concepts and comprehensive imaging evaluation. Radiographics. 2025;45:e240134. 10.1148/rg.240134. 40471833 10.1148/rg.240134

[31] Philippon MJ, Kuppersmith DA, Wolff AB, Briggs KK. Arthroscopic findings following traumatic hip dislocation in 14 professional athletes. Arthroscopy: The Journal of Arthroscopic & Related Surgery. 2009;25:169–74. 10.1016/j.arthro.2008.09.013. 19171277 10.1016/j.arthro.2008.09.013

[32] Sampatchalit S, Barbosa D, Gentili A, Haghighi P, Trudell D, Resnick D. Degenerative changes in the ligamentum teres of the hip: cadaveric study with magnetic resonance arthrography, anatomical inspection, and histologic examination. J Comput Assist Tomogr. 2009;33:927. 10.1097/RCT.0b013e318199d89e. 19940662 10.1097/RCT.0b013e318199d89e

[33] Roemer FW, Kwoh CK, Hannon MJ, Hunter DJ, Eckstein F, Fujii T, et al. What comes first? Arthritis Rheumatol Hoboken NJ. 2015;67:2085–96. 10.1002/art.39176. 10.1002/art.39176 PMC4519416 25940308

[34] Atukorala I, Kwoh CK, Guermazi A, Roemer FW, Boudreau RM, Hannon MJ, et al. Synovitis in knee osteoarthritis: a precursor of disease? Ann Rheum Dis. 2016;75:390–5. 10.1136/annrheumdis-2014-205894. 25488799 10.1136/annrheumdis-2014-205894 PMC4916836

